# Seroprevalence of human brucellosis in and around Jammu, India, using different serological tests

**DOI:** 10.14202/vetworld.2016.742-746

**Published:** 2016-07-17

**Authors:** H. K. Sharma, S. K. Kotwal, D. K. Singh, M. A. Malik, Arvind Kumar, Rajagunalan, M. Singh

**Affiliations:** 1 Division of Veterinary Public Health and Epidemiology, Faculty of Veterinary Sciences and Animal Husbandry, Sher-e-Kashmir University of Agricultural Sciences and Technology, Jammu, Jammu and Kashmir, India; 2 Division of Veterinary Public Health, Brucella Laboratory, Indian Veterinary Research Institute, Izzatnagar, Bareilly, Uttar Pradesh, India; 3 Division of Livestock Products Technology, Faculty of Veterinary Sciences and Animal Husbandry, Sher-e-Kashmir University of Agricultural Sciences and Technology, Jammu, Jammu and Kashmir, India

**Keywords:** brucellosis, humans, indirect enzyme-linked immunosorbent assay, Rose Bengal plate test, seroprevalence, standard tube agglutination test

## Abstract

**Aim:**

Brucellosis is a disease of zoonotic importance as it affects both human as well as animal’s health, and therefore, directly affects animal productivity and human efficiency. Therefore, a study was conducted to estimate the seroprevalence of brucellosis in humans in Jammu and surrounding areas.

**Materials and Methods:**

A total of 121 sera samples from humans occupied with professional related to animals were collected and tested for anti-*Brucella* antibodies by Rose Bengal plate test (RBPT), modified RBPT (mRBPT), standard tube agglutination test (STAT), and indirect enzyme-linked immunosorbent assay (I-ELISA). Sampling was done keeping in view with the occupation, sex, and age.

**Results:**

The overall seroprevalence of brucellosis recorded was 4.96%. The test-wise seroprevalence was 9.91% by RBPT, 9.91% by mRBPT, 9.09% by STAT, and 16.52% by I-ELISA. The prevalence of brucellosis was higher in >35-50 years age group compared to >20-35 years and >50-65 years. Sex-wise seroprevalence was higher in males than females. Taking I-ELISA as standard, the relative sensitivities of mRBPT, RBPT, and I-ELISA were in the order of mRBPT=RBPT>STAT. All the tests revealed high specificity values; however, among different serological tests, I-ELISA detected a maximum number of positive sera samples.

**Conclusions:**

The prevalence of brucellosis was found to be approximately 5%. The adult (>35-50 years) age male group was most vulnerable. The routine diagnosis of brucellosis involved the conventional serological tests, *viz*., RBPT and STAT, but each was associated with drawbacks which could give either false-positive or false-negative interpretation. Therefore, it is always recommended to use a battery of tests in the diagnosis of brucellosis.

## Introduction

Brucellosis is a zoonotic disease, and its persistence risk factors of existence are numerous and complex. Human brucellosis has serious public health consequences, caused by *Brucella melitensis* [1]. The disease is contracted mostly to those who live in proximity with animals. Human brucellosis is an infection with nonspecific symptoms initially, and often not detected in earlier phases [2]. Asymptomatic brucellosis infections mainly result from less frequent contact with *Brucella* and/or contact with low-virulence *Brucella*. Human brucellosis generally went unnoticed, undiagnosed by medical professionals owing to overlapping symptoms with other diseases. The eradication of Brucellosis in animals either through vaccination or destruction of infected animals is not feasible in country like India [3]. The prevention of human infection is primarily based on raising awareness, food-safety measures, personal, and occupational hygiene [4]. It affects people of all age groups of both sexes. Therefore, the control of disease in humans depends largely on the control of disease in animals [5].

Brucellosis, being zoonotic in nature, affects significantly both health and wealth. The routine diagnosis of brucellosis involves the conventional serological tests, *viz*., Rose Bengal plate test (RBPT) and standard tube agglutination test (STAT), but each is associated with certain drawbacks. Therefore, it is always recommended to use a battery of tests in the diagnosis of brucellosis including enzyme-linked immunosorbent assay (ELISA) which is reported to be a sensitive and specific test [6]. As per 2011 census, the human population of the study area was 15.30 lakhs with a total land area of 2342 km^2^. The livestock population of the study area was 7.683 lakhs. The documented data regarding human brucellosis in Jammu area are scarce and insufficient to conclude anything.

Thus, there is dire need to quantify the presence of disease in terms of prevalence in humans with underlying epidemiological features. Therefore, the present study aims at seroprevalence of human brucellosis in Jammu region using RBPT, modified RBPT (mRBPT), STAT and indirect ELISA (I-ELISA) serological tests.

## Materials and Methods

### Ethical approval

Appropriate consent was taken for collection of samples.

### Sample size

A total of 121 human sera samples were collected from Jammu district which is divided into four tehsils, namely, Akhnoor, Bishnah, Jammu, and Ranbir Singh Pora. The human sera sample was collected during study time of 6 months duration from the respective hospitals ethically and with the patients consent from those patients who suffered with swinging pyrexia of unknown region, arthralgia and had livestock holdings along with their age and sex. The sera samples were collected from randomlyfromJammu Medical College and Hospital (n=40), occupationally exposed groups,*viz*., associated staff working in Sheep Breeding Farm-Panthal, Jammu (n=7); slaughterhouse workers throughout Jammu districts from the Community Health Centers of Akhnoor, Bishnah, Jammu, and Ranbir Singh Pora (n=69); veterinarians and para-vets working under the Department of Animal Husbandry and sheep husbandry, Jammu, randomly (n=5).

### Blood collection

It was done by medical and para-medical staff by taking the consent of the patients using sterilized disposable syringes (5 ml) as per standard procedure and approved ethical guidelines. The serum samples were aliquoted and stored at −20°C for further use.

### Analytical tests

All the samples were subjected to RBPT, mRBPT, STAT, and I-ELISA tests. The RBPT and STAT tests were performed [7]. The ELISA was performed using smooth-lipopolysaccharide (S-LPS) extracted from *Brucella abortus* S 99. For the extraction, 5 g of lyophilized cells of *B. abortus* strain 99 was suspended in 170 ml of distilled water (DW) and heated to 66°C. An equal volume of phenol (90%; v/v) in DW, also heated to 66°C, was added, and the solution was stirred continuously for 20 min. It was then cooled to 4°C and centrifuged at 12,000*×g* for 20 min at 4°C. The phenol phase (bottom layer) was recovered and filtered through Whatman #1 to which three volumes of chilled methanol reagent was added. It was mixed thoroughly and left to precipitate at 4°C for 2 h. The precipitate was recovered by centrifugation at 12,000*×g* at 4°C and resuspended in the 80 ml of DW and centrifuged at 6000×g for 20 min. The pellet was resuspended in 80 ml of DW and stirred at 4°C overnight. The solution was then centrifuged at 10,000*×g* for 15 min at 4°C, and the supernatant was decanted. Another 80 ml of DW was added to the pellet, which was then stirred for 1 h and centrifuged as before. The two supernatants were pooled, filtered through membrane filter (0.3 µm) and 50-100 µg each of ribonuclease, deoxyribonuclease, and proteinase K were added. This mixture was incubated for 18 h at 20°C. It was reprecipitated with methanol and resuspended as above in 2 ml of DW. The solution was dialyzed extensively against DW until free of phenol. The resultant antigen was lyophilized, weighed, and resuspended in DW to give 1 mg LPS/ml. This was finally freeze dried in 1 ml volume and stored at 4°C for future use. For test proper, the LPS stock solution was thawed and vortexed. A 10 µl of stock LPS (1 mg/3 ml) was dispensed in the 10 ml coating buffer (carbonate/bicarbonate buffer; pH-9.6) and vortexed, and 100 µl per well was dispensed in flat bottom microtiter plates (Nunc). The plates were incubated at 4°C overnight. Next day, the plates were washed thrice using the phosphate buffer saline (PBS; 0.01M; pH-7.4) containing 0.05% Tween-20 (PBS-T). Before the final wash, 1:200 dilution of (predetermined) each test serum sample was prepared. After the final wash, 100 µl of each diluted serum was dispensed into the wells of microtiter plates, in duplicate and incubated for 1 h at 37°C. At the end of incubation, the plates were washed thrice with PBS-T as before and 100 µl of working dilution anti-species conjugate tagged with horse radish peroxidase (1.5:10,000) was dispensed into each well; and the plates were again incubated at 37°C for 1 h. The plates were washed thrice with PBS-T. After last wash, 100 µl of substrate solution containing 6 mg OPD (Sigma) and 4 µl H_2_O_2_ (30%) in 10 ml of substrate buffer (citrate buffer; pH-4.5) was dispensed into each well. Plates were kept in dark for 15 min for color development. After 15 min, the reaction was stopped by adding 50 µl of 3 M H_2_SO_4_ solution, and absorbance was measured at 492 nm in an ELISA reader (Bio-Rad Model 680) [8]. The samples were analyzed by mRBPT [9].

### Statistical analysis

The relative sensitivity and relative specificity of the test were calculated [10]. The kappa value, odd’s ratio, and relative risk were calculated using SPSS 6.0 software at 95% confidence interval.

## Results and Discussion

Human brucellosis (undulant fever) has assumed endemic proportions in many countries requiring a long course of the antibiotic therapy. However, prevention is still considered a better option, although no effective vaccine against human brucellosis is available till date. It has been postulated that human disease cannot be managed effectively without ensuring its prevention and control in animals. Therefore, information regarding the epidemiology of the disease in both animals and humans in a given region would be essential for the formulation and implementation of disease management strategies. Brucellosis is one of the most common occupational anthropozoonoses present worldwide resulting in huge economic losses and social burden in the developing countries. Besides direct public health implications, the prevalence of brucellosis poses as a potential barrier to international trade of animals and animal products [11].

The present study based on 121 human sera examination revealed an overall prevalence of 4.96% (Table-1), which was higher to that of 0.8% in Kashmir [12]. Migration of sheep from Kashmir to Jammu during winter and from Jammu to Kashmir is regular and usual phenomenon due to drastic change in the weather conditions. The Bakharwal and Gujjar community of the Jammu and Kashmir state do this migratory phenomenon every year, and they have the largest holding of these animals. The seroprevalence of brucellosis in humans (occupationally linked with handling of sheep and goats) using 121 serum samples, when tested by various tests, found to be 9.91% by RBPT, 9.91% by mRBPT, 9.09% by STAT, and 16.52% by I-ELISA. The seroprevalence rates were found to be highest by I-ELISA (26.09%), by RBPT (14.49%), and by STAT (10.14%) among slaughterhouse workers involved in sheep and goat slaughter as compared to other occupational groups, *viz*., general population, associated staff in sheep and goat rearing system (Table-2). However, all the serum samples (5) collected from veterinarians and para-veterinary staffs were found negative by all three tests, *viz*., RBPT, STAT and I-ELISA. The age-wise analysis of data in humans revealed the highest prevalence in the >20-35 years age group (9.59%, 10.96%, and 20.55% by RBPT, STAT, and I-ELISA, respectively), which could be due to their more occupational exposure to animal rearing. The results, however, differed from as reported the highest overall prevalence of 45.36% in the age group 41-50 years [13]. The determination of odd’s ratio, p value, and relative risk in humans is depicted in Table-3. It was clearly evident that higher prevalence in males (12.24%, 10.20%, and 20.41% by RBPT, STAT, and I-ELISA, respectively) as compared to females (0.0% by RBPT and I-ELISA and 4.35% by STAT). The presence of anti-*Brucella* antibodies in different serological test combinations in humans as depicted in Table-4. This was in accordance with the report of 98.6% prevalence in males as compared to 1.03% in females by I-ELISA [13]. The sensitivity (55.00%), as well as specificity (99.01%) of mRBPT, was found highest in case of humans as depicted in Table-5. In the present study, the sensitivity of RBPT was lower compared to other studies [14]. Further, in field conditions, the sensitivity of RBPT was known to vary from antigen to antigen preparation. In India, too, there were number of reports regarding lower sensitivity of RBPT [15,16]. STAT showed higher false-positive non-specific reactions which were lesser in RBPT due to acidic pH of the antigen [17]. Therefore, the use of STAT for *Brucella* diagnosis has been discouraged [18]. However, STAT detected the lesser number of positive samples than I-ELISA. Nevertheless, I-ELISA was observed to be better diagnostic test over RBPT and STAT and may be applied on a large scale for screening purposes for diagnosis of brucellosis in the country. The disease persists in the region lacking attention in its control. In the last few decades, disease drew scientific attention to reduce its prevalence using standardized conventional diagnostic methods [8]. Thus, further emphasis was laid for the development of serological tests of better sensitivity and specificity with a quick and economical diagnosis of brucellosis in man and animals. However, the serological tests differ in detection of various immunoglobulins (Ig) due to varying sensitivity to different *Brucella* infection, type, and purity of antigen besides variation in duration of incubation period during which test remains either positive or negative. To overcome such problems, new techniques have been developed to improve efficacy. I-ELISA was regarded as the gold standard test to detect bovine brucellosis; STAT and RBPT are traditional serological tests for rapid diagnosis of brucellosis in human and animals [19]. Serological tests varied in their sensitivity and specificity with respect to persistence of antibodies subsequent to infection. Numbers of serological tests were available for diagnosis of brucellosis of which RBPT, STAT, ELISA and complement fixation test have been used extensively to diagnose cases of brucellosis in livestock and humans. Complement fixation test was widely accepted and regarded as confirmatory test for diagnosis of brucellosis [8]. The other most incontrovertible diagnosis of brucellosis is isolation and identification of *Brucella*, often confronted with risk to laboratory workers. As such battery of serological tests, *viz*., RBPT, STAT, I-ELISA, etc., were recommended over complement fixation test and isolation of *Brucella* [20]. RBPT was a spot agglutination technique but can give both false positive as well as true positives. The sensitivity and specificity of the test reportedly vary with pH of the antigen, ambient temperature of antigen and test serum. The test was based on the detection of IgG_1_, IgG_2_, IgM, and IgA present in the test serum. An acidic pH of the buffer is reported to reduce the IgM activity. Most of the activity in this testis mediated through IgG isotypes as in complement fixation test. IgM reacts more efficiently with the antigen than IgG_1_ or IgG_2_ [21]. STAT measures total quantity of agglutinating antibodies but has the disadvantage of detecting post immunization agglutinins, sometime, to those caused by heterospecific antigens and cross-reacting antibodies. STAT measures IgM, IgG_1,_ IgG_2_, and IgA antibody types of which IgM type are more reactive than IgG_1_ and IgG_2._ Recently, ELISA (both, direct and indirect) has been introduced for diagnosis of brucellosis being as sensitive as radio immunoassay besides could be adopted for simple field screening procedures. The ELISA test has the advantage of giving clear-cut results with anti-complementary and hemolyzed sera. It also analyzes quantitative estimation of antibody concentration from a single dilution of serum. ELISA could be lead to the considerable saving of reagents, diluting equipment, and microtiter plates as compared to complement fixation test. Further, disease assumes importance as more than 70% of Indian population is rural being under threat of exposure constantly to the infected animals resulting in continuous transmission of disease to humans [22]. Therefore, the presence of disease in farmers, veterinarians, abattoir workers, and other occupationally exposed groups can never be questioned. Although, in past, few studies at limited scale have been conducted [23], no systematic study involving either in humans or animals were performed in Jammu region, thus ensuing the present study which was aimed to evaluate the prevalence of disease along with the underlying epidemiological features. The study also compared various routinely used serological tests, *viz*., RBPT, STAT, and I-ELISA in the diagnosis of brucellosis. However, no single test is in itself fullysatisfactory, and each test was associated with certain disadvantages [17]. Besides, due to the lack of reliability of tests, the seroprevalence has been calculated individually for each test.

**Table-1:** Seroprevalence of brucellosis among humans (n=121) in different places of Jammu as detected by RBPT, STAT, and I-ELISA.

Area (number of serum samples)	Tests (%)					

	RBPT		STAT		I-ELISA	
		
	Positive	Negative	Positive	Negative	Positive	Negative
General Population, Community Health Center-R.S. Pura (40)	0 (0.00)	40 (100.00)	2 (5.00)	38 (95.00)	0 (0.00)	40 (100.00)
Associated Staff of SBF-Panthal (7)	2 (28.57)	5 (71.43)	2 (28.57)	5 (71.43)	2 (28.57)	5 (71.43)
Slaughter House Workers-Jammu (69)	10 (14.49)	59 (85.51)	7 (10.14)	62 (89.86)	18 (26.09)	51 (73.91)
Veterinarians and para-vet staff-Jammu (5)	0 (0.00)	5 (100.00)	0 (0.00)	5 (100.00)	0 (0.00)	5 (100.00)
Total (n=121)	12 (9.91)	109 (90.08)	11 (9.09)	110 (90.90)	20 (16.52)	101 (83.47)

RBPT=Rose Bengal plate test, STAT=Standard tube agglutination test, I-ELISA=Indirect enzyme-linked immunosorbent assay

**Table-2:** Seroprevalence of brucellosis in humans (n=121).

Category	RBPT positive (%)	STAT positive (%)	I-ELISA positive (%)
Age wise			
>20-35 (young adults) (73)	7 (9.59)	8 (10.96)	15 (20.55)
>35-50 (middle aged) (31)	5 (16.13)	3 (9.68)	5 (16.13)
>50-65 (elderly adults) (17)	0 (0.00)	0 (0.00)	0 (0.00)
Sex wise			
Male (98)	12 (12.24)	10 (10.20)	20 (20.41)
Female (23)	0 (0.00)	1 (4.35)	0 (0.00)

RBPT=Rose Bengal plate test, STAT=Standard tube agglutination test, I-ELISA=Indirect enzyme-linked immunosorbent assay

**Table-3:** Determination of odd’s ratio, p value, and relative risk in humans (n=121)*.

Category	Odd’s ratio	p value	Relative risk
Age wise (years)			
>20-35	0.643 (0.098-4.219)	0.681	0.658 (0.108-3.992)
>35-50	3.107 (0.465-20.803)	0.174	2.903 (0.480-17.515)
>50-65	0.00 (0.00-6.035)	0.593	0.000 (0.000-5.217)
Sex wise			
Male (98)	Infinite (0.245-Infinite)	0.594	Infinite (0.269-Infinite)
Female (23)	0.00 (0.00-4.089)	0.594	0.00 (0.00-3.714)

* Using I-ELISA as diagnostic test. I-ELISA=Indirect enzyme-linked immunosorbent assay

**Table-4:** Presence of anti-*Brucella* antibodies in different serological test combinations in humans (n=121).

Test	1	2	3	4	5	6	7	8
RBPT	-	+	-	-	+	-	+	+
STAT	-	-	+	-	+	+	-	+
I-ELISA	-	-	-	+	-	+	+	+
General Population, Community Health Centre-R.S. Pura (40)	38	0	2	0	0	0	0	0
Associated staff of SBF-Panthal (7)	5	0	0	0	0	0	0	2
Slaughter house Workers-Jammu (69)	49	0	1	8	1	1	5	4
Veterinarians and para-veterinarian staff-Jammu (5)	5	0	0	0	0	0	0	0
Total (n=121)	97	0	3	8	1	1	5	6

RBPT=Rose Bengal plate test, STAT=Standard tube agglutination test, I-ELISA=Indirect enzyme-linked immunosorbent assay

**Table-5:** Statistical analysis of RBPT, STAT and mRBPT in diagnosis of brucellosis in humans (n=121)*.

Test	kappa value	Sensitivity	Specificity
RBPT	0.643^b^ (0.399-0.711)	55.00^b^ (32.04-6.17)	99.00^b^ (93.81-99.94)
STAT	0.379^a^ (0.136-0.566)	35.00^a^ (16.30-9.05)	96.03^a^ (89.59-98.72)
mRBPT	0.643^b^ (0.399-0.711)	55.00^b^ (32.04-6.17)	99.01^b^ (93.81-99.95)

* I-ELISA as the standard, Values in a column with different superscripts are significantly different (p<0.05). mRBPT=Modified Rose Bengal plate test, STAT=Standard tube agglutination test, I-ELISA=Indirect enzyme-linked immunosorbent assay

## Conclusions

The routine diagnosis of brucellosis involves the conventional serological tests, *viz*., RBPT and STAT, but each is associated with certain drawbacks. The prevalence of brucellosis was found to be on higher side. The young age male group was most vulnerable. Accordingly, it is always recommended to use a battery of tests in human brucellosis diagnosis.

## Authors’ Contributions

SKK, DKS, and MAM designed the research program, HKS conducted the research work, R and MS helped in conduct of research work and statistical analysis, AK and HKS prepared the manuscript. All authors read and approved the final manuscript.
